# Logistic Regression Analysis Factors Affecting Sperm Motility and Abnormal Sperm Morphology in Boars

**DOI:** 10.3390/ani9121004

**Published:** 2019-11-20

**Authors:** Yinghui Wu, Chao Wang, Jiajian Tan, Hong-kui Wei, Haiqing Sun, Jian Peng

**Affiliations:** 1 Department of Animal Nutrition and Feed Science, College of Animal Science and Technology, Huazhong Agricultural University, Wuhan 430070, China; academy_wuyinghui@163.com (Y.W.); academy_wangchao@163.com (C.W.); weihongkui@mail.hzau.edu.cn (H.-k.W.); 2 YangXiang Joint Stock Company, Guigang 537000, China; tjjggyx@sina.com (J.T.); fbsw_027@163.com (H.S.); 3 The Cooperative Innovation Center for Sustainable Pig Production, Wuhan 430070, China

**Keywords:** abnormal sperm morphology, boar, element, logistic regression, sperm motility

## Abstract

**Simple Summary:**

Reduced sperm motility and morphological abnormalities have significant negative impacts on conception rates in sows and are important indexes of semen. The identification of factors that influence sperm motility and morphology will improve boar fertility in commercial herds. On the basis of analysis of 5042 ejaculates from 385 boars using a logistic regression model, we found that serum Cu excess, serum Fe deficiency, and Pb presence in seminal plasma were risk factors for poor semen quality in boars. More importantly, the presence of seminal plasma Pb had more serious effect on the probability of abnormal sperm morphology than serum Cu excess and serum Fe deficiency. In addition, Yorkshire and Landrace boars had higher sperm motility and lower abnormal sperm morphology than Duroc boars. The difference in serum and seminal plasma elements among boars with different semen qualities may become a guide for regulating these elements used in boar diet. The highly predictive values of serum Cu, Fe, and seminal plasma Pb could be used in the future as an additional tool in semen quality evaluation.

**Abstract:**

Logistic regression models, including variables of boar breed, age, serum, and seminal plasma elements, were used to identify the influencing factors of sperm motility and morphology in this study. Sperm motility degree was classified as grade 0: ≤85% and grade 1: >85%. Abnormal sperm morphology was classified as grade 0: ≤10%, grade 1: 10–20%, and grade 2: >20%. Element concentration of 385 boars was detected by inductively coupled plasma mass spectrometry. Results showed that boars with serum Cu ≥ 2.5 mg/L had lower sperm motility (odds ratio (OR): 0.496; 95% confidence interval (CI): 0.285–0.864) and higher abnormal sperm morphology (OR: 2.003; 95% CI: 1.189–3.376) than those with serum Cu ≤ 2.0 mg/L. Boars with serum Fe ≥ 1.5 mg/L had lower abnormal sperm morphology than those with serum Fe ≤ 1.0 mg/L (OR: 0.463; 95% CI: 0.255–0.842). The presence of Pb in seminal plasma increased abnormal sperm morphology. The probability of abnormal sperm morphology >20% from boars with seminal plasma Pb increased with a range of 5.78–15.30% than that from boars without seminal plasma Pb among three breeds. In conclusion, serum Cu excess, serum Fe deficiency, and seminal plasma Pb are risk factors for poor semen quality in boars.

## 1. Introduction

Boars are recognized as a significant source of variation with regard to success in vivo [1] and in vitro [2] fertilization in swine. Boar sperm motility and morphology, which relate to the sow conception rate, are important indexes for evaluating semen quality [3,4]. Therefore, predicting the probability of boars with poor sperm motility and morphology according to identified potential influencing factors could improve the semen quality of individual boar and economic benefit of commercial herds.

Cu, Fe, Zn, Se, and other nutrient elements are components of cells and essential cofactors of bioactive molecules present in the male reproductive system. However, their excessive deposition may be harmful to male fertility above certain levels [5,6]. Nutrient element concentrations in serum or seminal plasma affect the semen quality of boars and other male animals [7,8,9]. In addition, Pb and Cd are highly toxic for mammals and are negatively correlated with male reproductive parameters [10,11]. Our previous studies also found that the semen quality of Duroc boars was mainly affected by serum Cu, Fe, Mn, and seminal plasma Cd, whereas that of Yorkshire boars was mainly affected by serum Cu, Fe, Se, and seminal plasma Pb [12,13]. Nevertheless, artificial insemination studs consist of boars with different breeds and ages, which affect the relationship between elements in serum or seminal plasma and semen quality in commercial herds [14,15,16]. Furthermore, producers may care more about the proportion of boars with poor semen quality accompanied by risk factors in production conditions.

Generally, logistic regression is frequently used in medical research and well suited for testing hypotheses about relationships between one categorical outcome variable and several categorical or continuous predictor variables [17,18]. In pig production, Farzan et al. found that management factors, including feed types and breeding methods, were risk factors for shedding Salmonella with or without antimicrobial resistance by using a multinomial logistic regression model [19]. However, these studies were unable to further explore the extent of these risk factors. In addition, few studies have predicted the probability of boars with poor semen quality according to the identified potential factors in commercial herds.

Therefore, the present study aimed to (1) explore risk factors affecting sperm motility and abnormal sperm morphology in the aspects of boar breed, age, and elements in serum and seminal plasma and (2) predict the probability of boars with poor sperm motility and morphology according to the identified risk factors by using logistic regression model in commercial herds.

## 2. Materials and Methods

### 2.1. Herds and Animals

This study was conducted on an artificial insemination center located in Southern China. All animal handling protocols were approved by the Scientific Ethic Committee of Huazhong Agricultural University (approval permit number HZAUSW-2016-011) and were conducted in accordance with the National Research Council’s Guide for the Care and Use of Laboratory Animals.

A total of 385 boars (166 Duroc, 107 Landrace, and 112 Yorkshire boars) aged 9–55 months were selected. The experimental boars were housed within the same building in individual crates (0.79 m × 2.40 m) with concrete-slotted floors (90 mm solid width, 23 mm slots). An automated production system, which included a positive pressure ventilation, automatic feeding, and heating systems, was used to control the indoor environment (Automated Production Systems, 1004 E. Illinois St. Assumption, IL 62510, USA). The experimental boars were limit fed a commercially prepared corn and soybean meal-based diet at a rate of 2.5 kg/day with ad libitum access to water. The nutrition level of boar diet was as follows: 2.30 Mcal/kg net energy, 14.25% crude protein, 2.50% crude fat, 5.54% crude ash, 3.46% crude fiber, 0.75% Lysine, 0.32% Methionine, 0.55% Threonine, 0.15% Tryptophan, 0.82% total Ca, and 0.77% total phosphate. Premix provided the following minerals per kilogram: 17 mg of Cu, 160 mg of Fe, 140 mg of Zn, 50 mg of Mn, 0.50 mg of I, 0.50 mg of Se, and 0.22 mg of Cr.

### 2.2. Semen Quality Data Collection

Boars were trained to mount artificial sows and semen was collected three times every two weeks. Sperm-rich ejaculate fractions were collected by the gloved-hand technique during ejaculation and the gelatinous fraction was strained from the ejaculate through four layers of cotton-mesh gauze. The sperm motility and abnormal sperm morphology of each ejaculation of experimental boars were evaluated from June to August in 2016. The methods for evaluating boar semen quality were described in our previous study [13]. Briefly, semen was diluted 1:1 (*v*/*v*) with isothermal Beltsville Thawing Solution (Jin Li Livestock Equipment Co., LTD, Wuhan, China). Approximately 10 μL of semen sample was placed in a pre-warmed (37 °C) microscope slide and then the sperm motility was analyzed by computer-aided sperm analysis system (ML-210JZ, Nanning SongJing TianLun Biological Technology Co., LTD, Nanning, China). Abnormal sperm, including head defects, neck, and mid-piece defects, tail defects, and excess residual cytoplasm, were counted using the eosin stain-dried method. At least 200 spermatozoa were evaluated using bright-field optical microscope (ML-210JZ, Nanning SongJing TianLun Biological Technology Co., LTD, Nanning, China) at 400× [20]. In this study, the semen quality data of 5042 ejaculates from 385 boars were recorded and the mean values of sperm motility and abnormal sperm morphology were calculated for each boar.

### 2.3. Element Determination in Serum and Seminal Plasma Samples

Serum and seminal plasma samples from 385 boars were collected to determine the concentrations of 9 elements, including Ca, Mg, Cu, Fe, Zn, Mn, Se, Pb, and Cd, by using inductively coupled plasma mass spectrometry (Agilent 7900, Agilent Technologies, Tokyo, Japan). Blood samples were collected by venipuncture after an overnight fast of at least 12 h. Blood was allowed to clot and serum was recovered after centrifugation at 1500× *g* for 10 min at room temperature. Semen samples were collected at the same time as blood samples and further centrifugation was performed to obtain seminal plasma samples. Serum and seminal plasma samples were stored at −80 °C until trace element analysis. The methods for determining the content of serum and seminal plasma elements were described in our previous study [12].

### 2.4. Statistical Analysis

Multiple variables of boar breed, age, serum, and seminal plasma elements were evaluated using univariable and multivariate logistic regression analysis in this study. According to the distribution characteristics of boar sperm motility data (Table 1), boars with a sperm motility of <70% were very few (2.34%; n = 9) during the examination period. Therefore, we divided boars into grade 0: ≤85% (158, 41.04%) and grade 1: >85% (227, 58.96%) according to the actual condition. The abnormal sperm morphology was classified as grade 0: ≤10% (109, 28.31%), grade 1: 10–20% (209, 54.28%), and grade 2: >20% (67, 17.40%) on the basis of the distribution characteristics of abnormal sperm morphology (Table 2). First, variables were screened on the basis of the measures of multicollinearity (correlation coefficient |r| >0.7). Thereafter, a univariable analysis was performed for each independent variable and independent variables with *p*-value < 0.1 in univariable analysis were included into the multivariable logistic regression. Finally, the potential risk factors were identified within the multivariable analysis on the basis of the forward stepwise selection by using a *p*-value < 0.05. The regression coefficients were expressed as odds ratios (OR) with 95% confidence intervals (CI). All analytical procedures were performed with SAS for data analysis (version 9.2; SAS Inst. Inc., Cary, NC, USA). The model is formulated as follows:

Logit (P) = β_0_ + β_a_ A + β_b_ B + β_c_ C + β_d_ D + β_e_ E + β_f_ F + β_g_ G + β_h_ H

where β_0_ was the intercept. A, B, C, D, E, F, G, and H represented breed, age, serum Cu, serum Fe, serum Mn, serum Se, seminal plasma Pb, and seminal plasma Cd, respectively. β_a_ (a included 3 dummy variables), β_b_ (b included 4 dummy variables), β_c_ (c included 3 dummy variables), β_d_ (d included 3 dummy variables), β_e_ (e included 3 dummy variables), β_f_ (f included 3 dummy variables), β_g_ (g included 3 dummy variables), and β_h_ (h included 3 dummy variables) were the slope for each dummy variable.

## 3. Results

### 3.1. Potential Risk Factors of Boar Sperm Motility and Abnormal Sperm Morphology

On the basis of the results of univariate logistic regression analysis, independent variables with *p*-value < 0.10 were included into the multiple logistic regression model so as not to omit the potential influencing variables. Results showed that the sperm motility was influenced by boar breed, serum Cu and Fe, and seminal Pb (Table 3). The abnormal sperm morphology was influenced by boar breed, serum Cu and Fe, and seminal plasma Pb and Cd contents (Table 4).

### 3.2. Prediction Probability of Boars with Poor Sperm Motility by Using a Binary Logistic Regression Model

Results of the logistic regression analysis of the selected factors in the sperm motility model are summarized in Table 5 and Table 6. The sperm motility of boars was significantly influenced by breed and serum Cu concentration in the study (*p* < 0.05; Table 5). Yorkshire (OR: 3.060; 95% CI: 1.762 to 5.315) and Landrace boars (OR: 1.976; 95% CI: 1.218 to 3.205) had higher sperm motility than Duroc boars. However, boars with serum Cu ≥ 2.5 mg/L had lower sperm motility than those with serum Cu ≤ 2.0 mg/L (OR: 0.496; 95% CI: 0.285 to 0.864). On the basis of the estimated coefficients of independent variables in Table 5, the logistic regression model of sperm motility was presented as follows: Logit (P_motility > 85%_) = log (odds of motility >85%) = 0.258 + 1.119 × breed (Yorkshire) + 0.681 × breed (Landrace) − 0.700 × serum Cu (≥2.5 mg/L). For example, the probability of sperm motility >85% was 56.41% in Duroc boars with serum Cu < 2.5 mg/L (log (odds of motility >85%) = 0.258; odds = exp (0.258) = 1.294; P_motility > 85%_ = odds/(1 + odds) = 0.5641). Similarly, we calculated the predictive probability of sperm motility ≤85% and sperm motility >85% among different breeds with varying serum Cu levels. The predictive probability of sperm motility >85% decreased with a range from 13.54% to 17.27% in boars with serum Cu ≥ 2.5 mg/L compared with that in boars with serum Cu ≤ 2.0 mg/L among three breeds (Table 6).

### 3.3. Prediction Probability of Boars with High Abnormal Sperm Morphology by Using the Ordered Logistic Multiple Regression Model

As shown in Table 7, abnormal sperm morphology was influenced by breed, serum Cu and Fe, and seminal plasma Pb concentration (*p* < 0.05). Yorkshire (OR: 0.328; 95% CI: 0.194 to 0.553) and Landrace boars (OR: 0.236; 95% CI: 0.145 to 0.386) had lower abnormal sperm morphology than Duroc boars. In terms of element, boars with serum Cu ≥ 2.5 mg/L had higher abnormal sperm morphology than those with serum Cu ≤ 2.0 mg/L (OR: 2.003; 95% CI: 1.189 to 3.376). Boars with serum Fe ≥ 1.5 mg/L had lower abnormal sperm morphology than those with serum Fe ≤ 1.0 mg/L (OR: 0.463; 95% CI: 0.255 to 0.842). Furthermore, boars with seminal plasma Pb between 0 μg/L and 10 μg/L (OR: 1.666; 95% CI: 1.074 to 2.584) and ≥10 μg/L (OR: 2.204; 95% CI: 1.132 to 4.291) had a higher abnormal sperm morphology than those without seminal plasma Pb.

On the basis of the estimated coefficients of independent variables in Table 7, the two logistic regression models of abnormal sperm morphology were presented as follows: Equation (1): Logit (P_abnormal > 10%_) = log (odds of abnormal >10%) = 1.426 − 1.116 × breed (Yorkshire) − 1.443 × breed (Landrace) + 0.695 × serum Cu (≥2.5 mg/L) − 0.769 × serum Fe (≥1.5 mg/L) + 0.511 × seminal Pb (0 − 10 μg/L) + 0.790 × seminal Pb (≥10 μg/L); Equation (2): Logit (P_abnormal > 20%_) = log (odds of abnormal >20%) = −1.418 − 1.116 × breed (Yorkshire) − 1.443 × breed (Landrace) + 0.695 × serum Cu (≥2.5 mg/L) − 0.769 × serum Fe (≥1.5 mg/L) + 0.511 × seminal Pb (0 − 10 μg/L) + 0.790 × seminal Pb (≥10 μg/L). For example, the probability of abnormal sperm morphology ≤10% was 19.37% in Duroc boars with serum Cu ≤ 2.0 mg/L (log (odds) = 1.426; P_abnormal > 10%_ = odds/(1 + odds) = 0.8063; P_abnormal ≤ 10%_= 1 − P_abnormal > 10%_ = 0.1937). Table 8 summarizes the predictive probability of the abnormal sperm morphology ≤10% and abnormal sperm morphology >20% among different breeds with varying element concentrations. The probabilities of abnormal sperm morphology >20% in Duroc, Yorkshire, and Landrace boars were 23.55%, 9.41%, and 7.00%, respectively. Furthermore, Duroc, Yorkshire, and Landrace boars with serum Cu ≥ 2.5 mg/L had a 13.17%, 6.37%, and 4.87% higher probability of abnormal sperm morphology >20% than those with serum Cu ≤ 2.0 mg/L, respectively. In addition, Duroc, Yorkshire, and Landrace boars with serum Fe ≥ 1.5 mg/L had a 9.41%, 3.80%, and 2.83% lower probability of abnormal sperm morphology >20% than those with serum Fe ≤ 1.0 mg/L, respectively. More importantly, Duroc, Yorkshire, and Landrace boars with seminal plasma Pb ≥ 10 μg/L had a 15.30%, 7.53%, and 5.78% higher probability of abnormal sperm morphology >20% than those without seminal plasma Pb, respectively.

## 4. Discussion

This study explored and predicted risk factors affecting sperm motility and abnormal sperm morphology by using a multivariate regression model in a commercial herd. We found that boar breed, serum Cu and Fe, and seminal plasma Pb concentration had significant impacts on these two parameters. Sperm motility tended to increase with the decrease of serum Cu. The abnormal sperm morphology tended to decrease with the decrease of serum Cu and seminal plasma Pb and increase of serum Fe.

The logistic regression model is generally used to identify the influencing factors and predict the probability of categorical dependent variables. According to data distribution characteristics and production criteria of boar sperm motility and abnormal sperm morphology in commercial herds, these two parameters were divided into categorical variables and appropriate logistic regression models were used to predict the probability of boars with poor sperm motility and morphology in this study. We found that the probability of poor sperm motility (≤85%) varied from 20.15% to 60.86%, and that of higher abnormal sperm morphology (>20%) varied from 2.58% to 34.80% among different boar breeds and element concentrations. This finding suggests that controlling risk factors identified in this study may have a great value in improving boar semen quality in commercial herds.

Boar breed was confirmed to have a significant influence on sperm motility and abnormal sperm morphology in this study. Results showed that Duroc boars had a higher risk of poor sperm motility (≤85%) and higher abnormal sperm morphology (>20%) compared with Yorkshire and Landrace boars. Smital (2009) [15] found that the sperm motility and sperm output of Duroc boars were significantly lower than those of Yorkshire and Landrace boars. In addition, the abnormal sperm morphology of Duroc boars was significantly higher than that of Yorkshire and Landrace boars [21]. However, the relationship between boar age and sperm motility and abnormal sperm morphology was not significant in the present study. This may be because age mainly affects semen volume, sperm concentration, and the total sperm number of boars but has no significant effect on sperm motility and abnormal sperm morphology [16].

Cu is necessary for the maintenance of normal spermatogenesis and sperm function as it plays a dominant role in diverse proteins, such as cytochrome oxidase and cytoplasmic superoxide dismutase [22,23]. However, an excess of Cu has a negative impact on cell morphology and function because it can enhance the production of free radicals [24,25]. Our study also demonstrates that an excess of the serum Cu had a negative influence on sperm motility and morphology. Similar to these results, a decline of progressive motility along with the increase in serum Cu was found in Li’s study [7]. This phenomenon may be attributed to the liver and reproductive organs having greater ability to accumulate Cu, which leads to excess Cu accumulation in the reproductive organs and results in decreased mitochondrial activity and adenosine triphosphate production [26]. Fe plays an important role in the essential ecophysiological component of cells and tissues in the male reproductive system [5]. In this study, abnormal sperm morphology tended to decrease with the increase of serum Fe content. This result indicates that the serum Fe concentration positively affected semen quality and it may be insufficient in boars with a high abnormal sperm morphology (>20%). A lack of Fe affects the activity of Fe-dependent or Fe-containing enzymes, which increases lipid peroxidation in boar spermatozoa [27]. Therefore, inadequate serum Fe leads to poor semen quality in boars, which is confirmed from our previous findings in Duroc and Yorkshire boars [12,13]. The effect of increased serum Cu concentration on the occurrence probability of abnormal sperm morphology >20% was higher than that of increased serum Fe concentration. This finding suggests that the toxic effects of element excess might outweigh the effects of element deficiency.

Evidence from animal and human studies suggests that toxic element Pb and Cd may have adverse impacts on male reproductive health at relatively low levels [10,28,29]. The total sperm number, sperm motility and morphology [30,31], and sperm metabolism [32] are damaged by exposure to Pb. In the present study, toxic element Pb in seminal plasma increased the probability of abnormal sperm. More importantly, boars with a maximum seminal plasma Pb concentration ≥10 μg/L increased the probability of abnormal sperm (>20%) by 15.30% compared with those without seminal plasma Pb. Pb compounds can induce reactive oxygen species (ROS) production, which leads to lipid peroxidation and the functional damage of sperm [30]. This result indicates that sperm exposed to toxic elements may suffer more damage than that under deficiency and excess of nutrient elements. Eghbali et al. also claimed that seminal plasma Pb had an adverse impact on motility and viability of spermatozoa after ejaculation in water buffalo [28]. A number of studies have documented that environmental or medicinal chemicals could present in semen and impact semen quality [33,34]. Therefore, Pb concentrations in boar diet and drinking water were also analyzed in the present study. We found that the Pb content in the diet was 0.14 mg/kg, whereas it was not detected in the drinking water. We speculate that the presence of Pb in the diet may be the provenance of Pb in boar seminal plasma. Although the Pb content in boar diet in this study was lower than the feed hygiene standards of China (GB/T13078-2001), Pb has a much longer half-life and may lead to biological accumulation in tissues and organs [35]. Toxic element Cd has been confirmed to be related to poor semen quality and DNA damage [29,36]. Similar to Pb, Cd could also result in ROS production, excessive protein oxidation, lipid peroxidation, and cell death [37,38]. However, different from the results in our previous study, the effect of seminal plasma Cd on semen quality was not significant in this study [12]. The reasons may be because seminal plasma Cd concentrations of 45.45% boars in this study were lower than the detection limit and the extremely low Cd level affects the analysis results between Cd and sperm quality parameters.

## 5. Conclusions

Serum Cu excess, serum Fe deficiency, and the presence of Pb in seminal plasma are risk factors for poor semen quality in boars. More importantly, sperm exposed to toxic element Pb may suffer more damage than that under deficiency and excess of nutrient elements. Hence, producers should pay more attention to the reasonable addition of nutrient elements and strictly control the pollution of toxic elements in boar diet. In addition, serum Cu and Fe and seminal plasma Pb concentration may be used in the future as an additional tool in semen quality evaluation and prediction.

## Figures and Tables

**Table 1:** Data distribution of mean sperm motility at three months in experimental boars.

Item	Sperm Motility						Total
	≤70%	70% < X ≤ 80%	80% < X ≤ 85%	85% < X ≤ 87.5%	87.5% < X ≤ 90%	>90%	
Sample size	9	44	105	115	97	15	385
Rate	2.34%	11.43%	27.27%	29.87%	25.19%	3.90%	100%

**Table 2:** Data distribution of mean abnormal sperm morphology at three months in experimental boars.

Item	Abnormal Sperm Morphology						Total
	≤5%	5% < X ≤ 10%	10% < X ≤ 15%	15% < X ≤ 20%	20% < X ≤ 25%	>25%	
Sample size	2	107	137	72	38	29	385
Rate	0.52%	27.79%	35.58%	18.70%	9.87%	7.53%	100%

**Table 3:** The potential influencing factors and univariate analysis results of the sperm motility (n = 385).

Item	Motility 0 (n = 158)	Motility 1 (n = 227)	*p*-Value	OR (95%CI) ^1^
Breed				
Duroc (ref) ^2^	87 (55.06%) ^3^	79 (34.80%)	-	-
Yorkshire	27 (17.09%)	68 (29.96%)	<0.01 *	2.773 (1.616–4.757)
Landrace	44 (27.85%)	80 (35.24%)	<0.01 *	2.002 (1.242–3.228)
Age				
≤12 mo (ref)	20 (12.66%)	37 (16.30%)	-	-
13–18 mo	51 (32.28%)	61 (26.87%)	0.195	0.647 (0.335–1.250)
19–31 mo	64 (40.51%)	93 (40.97%)	0.453	0.785 (0.418–1.475)
≥32 mo	23 (14.56%)	36 (15.86%)	0.664	0.846 (0.398–1.800)
Serum Cu				
≤2.0 mg/L (ref)	43 (27.22%)	84 (37.00%)	-	-
2.0–2.5 mg/L	65 (41.14%)	88 (38.77%)	0.141	0.693 (0.426–1.129)
≥2.5 mg/L	50 (31.65%)	55 (24.23%)	0.034 *	0.563 (0.331–0.957)
Serum Fe				
≤1.0 mg/L (ref)	48 (30.38%)	48 (21.15%)	-	-
1.0–1.5 mg/L	78 (49.37%)	129 (56.83%)	0.044 *	1.654 (1.014–2.697)
≥1.5 mg/L	32 (20.25%)	50 (22.03%)	0.143	1.562 (0.860–2.840)
Serum Mn				
0 μg/L (ref)	44 (27.85%)	99 (43.61%)	-	-
0–10 μg/L	52 (32.91%)	60 (26.43%)	0.184	0.655 (0.351–1.222)
≥10 μg/L	62 (39.24%)	68 (29.96%)	0.503	0.814 (0.445–1.488)
Serum Se				
≤150 μg/L (ref)	29 (18.35%)	36 (15.86%)	-	-
150–250 μg/L	90 (56.96%)	139 (61.23%)	0.670	1.142 (0.620–2.103)
≥250 μg/L	39 (24.68%)	52 (22.91%)	0.974	1.011 (0.506–2.022)
Seminal Pb				
0 μg/L (ref)	49 (31.01%)	79 (34.80%)	-	-
0–10 μg/L	85 (53.80%)	125 (55.07%)	0.689	0.912 (0.581–1.431)
≥10 μg/L	24 (15.19%)	23 (10.13%)	0.100 *	0.594 (0.303–1.166)
Seminal Cd				
0 μg/L (ref)	67 (42.41%)	108 (47.58%)	-	-
0–0.5 μg/L	64 (40.51%)	89 (39.21%)	0.513	0.863 (0.554–1.343)
≥0.5 μg/L	27 (17.09%)	30 (13.22%)	0.226	0.689 (0.377–1.259)

^1^ OR = odds ratio; CI = confidence interval. ^2^ Ref = reference. ^3^ 87 (55.06%): Outside the parenthesis is the sample size, n = 87; the proportion of the sample size in the group is shown in parentheses, which is 55.06%. * *p* < 0.10.

**Table 4:** The potential influencing factors and univariate analysis results of the abnormal sperm morphology (n = 385).

Item	Abnormal 0 (n = 109)	Abnormal 1 (n = 209)	Abnormal 2 (n = 67)	*p*-Value	OR (95%CI) ^1^
Breed					
Duroc (ref) ^2^	19 (17.43%) ^3^	107 (51.20%)	40 (59.70%)	-	-
Yorkshire	33 (30.28%)	50 (23.92%)	12 (17.91%)	<0.01 *	0.345 (0.208–0.574)
Landrace	57 (52.29%)	52 (24.88%)	15 (22.39%)	<0.01 *	0.230 (0.142–0.373)
Age					
≤12 mo (ref)	14 (12.84%)	34 (16.27%)	9 (13.43%)	-	-
13–18 mo	23 (21.10%)	64 (30.62%)	25 (37.31%)	0.341	1.350 (0.729–2.500)
19–31 mo	50 (45.87%)	83 (39.71%)	24 (35.82%)	0.428	0.789 (0.440–1.416)
≥32 mo	22 (20.18%)	28 (13.40%)	9 (13.43%)	0.248	0.661 (0.328–1.334)
Serum Cu					
≤2.0 mg/L (ref)	40 (36.70%)	75 (35.86%)	12 (17.91%)	-	-
2.0–2.5 mg/L	49 (44.95%)	70 (33.49%)	34 (50.75%)	0.177	1.366 (0.868–2.151)
≥2.5 mg/L	20 (18.35%)	64 (30.62%)	21 (31.34%)	<0.05 *	1.900 (1.149–3.141)
Serum Fe					
≤1.0 mg/L (ref)	19 (17.43%)	51 (24.40%)	26 (38.81%)	-	-
1.0–1.5 mg/L	56 (51.38%)	123 (58.85%)	28 (41.79%)	<0.05 *	0.547 (0.341–0.878)
≥1.5 mg/L	34 (31.19%)	35 (16.75%)	13 (19.40%)	<0.01 *	0.363 (0.204–0.647)
Serum Mn					
0 μg/L (ref)	42 (38.53%)	82 (39.23%)	18 (26.87%)	-	-
0–10 μg/L	27 (24.77%)	62 (29.67%)	23 (34.33%)	0.287	1.387 (0.759–2.533)
≥10 μg/L	40 (36.70%)	65 (31.10%)	26 (38.81%)	0.871	1.049 (0.589–1.868)
Serum Se					
≤150 μg/L (ref)	19 (17.43%)	36 (17.22%)	10 (14.93%)	-	-
150–250 μg/L	63 (59.80%)	128 (61.24%)	38 (56.72%)	0.827	1.067 (0.597–1.905)
≥250 μg/L	27 (24.77%)	45 (21.53%)	19 (28.36%)	0.719	1.129 (0.584–2.182)
Seminal Pb					
0 μg/L (ref)	48 (44.04%)	59 (28.23%)	21 (31.34%)	-	-
0–10 μg/L	52 (47.71%)	122 (58.37%)	36 (53.73%)	<0.05 *	1.524 (1.007–2.331)
≥10 μg/L	9 (8.26%)	28 (13.40%)	10 (14.93%)	<0.05 *	2.012 (1.052–3.848)
Seminal Cd					
0 μg/L (ref)	57 (52.29%)	93 (44.50%)	25 (37.33%)	-	-
0–0.5 μg/L	38 (34.86%)	84 (40.19%)	31 (45.75%)	0.134	1.583 (0.811–2.721)
≥0.5 μg/L	14 (12.84%)	32 (15.31%)	11 (16.93%)	0.243	1.410 (0.792–2.511)

^1^ OR = odds ratio; CI = confidence interval. ^2^ Ref = reference. ^3^ 19 (17.43%): Outside the parenthesis is the sample size, n = 19; the proportion of the sample size in the group is shown in parentheses, which is 17.43%. * *p* < 0.10.

**Table 5:** The factors that influence the sperm motility were extracted by forward selection in binary logistic regression analysis (n = 385).

Variable	Estimate	SE	Odds Ratio	95% CI ^1^		*p*-Value
				Lower 95%	Upper 95%	
Intercept	0.258	0.217	-	-	-	0.234
Breed						
Duroc (ref) ^2^	0 ^3^	0	0	0	0	-
Yorkshire	1.119	0.282	3.060	1.762	5.315	<0.01
Landrace	0.681	0.247	1.976	1.218	3.205	<0.01
Serum Cu						
≤2.0 mg/L (ref)	0	0	0	0	0	-
2.0–2.5 mg/L	−0.445	0.255	0.641	0.389	1.057	0.081
≥2.5 mg/L	−0.700	0.283	0.496	0.285	0.864	<0.05

^1^ CI = confidence interval. ^2^ Ref = reference. ^3^ “0” represented the reference category for each explanatory variable.

**Table 6:** Prediction of the occurrence probability of boar sperm motility less than or greater than 85% under different breeds and serum Cu concentrations.

Breed	Subgroups	P (Motility ≤ 85%)	P (Motility > 85%)
Duroc	Serum Cu ≤ 2.0 mg/L	43.59%	56.41%
	Serum Cu ≥ 2.5 mg/L	60.86%	39.14%
	Mean value	52.23%	47.78%
Yorkshire	Serum Cu ≤ 2.0 mg/L	20.15%	79.85%
	Serum Cu ≥ 2.5 mg/L	33.69%	66.31%
	Mean value	26.92%	73.08%
Landrace	Serum Cu ≤ 2.0 mg/L	28.11%	71.89%
	Serum Cu ≥ 2.5 mg/L	44.05%	55.95%
	Mean value	36.08%	63.92%

**Table 7:** The factors that influence the abnormal sperm morphology were extracted by forward selection in ordered logistic regression analysis (n = 385).

Variable	Estimate	SE	Odds Ratio	95% CI ^1^		*p*-Value
				Lower 95%	Upper 95%	
Intercept 2	−1.418	0.309	-	-	-	<0.01
Intercept 1	1.426	0.312	-	-	-	<0.01
Breed						
Duroc (ref) ^2^	0 ^3^	0	0	0	0	-
Yorkshire	−1.116	0.267	0.328	0.194	0.553	<0.01
Landrace	−1.443	0.251	0.236	0.145	0.386	<0.01
Serum Cu						
≤2.0 mg/L (ref)	0	0	0	0	0	-
2.0–2.5 mg/L	0.457	0.238	1.579	0.989	2.519	0.056
≥2.5 mg/L	0.695	0.266	2.003	1.189	3.376	<0.01
Serum Fe						
≤1.0 mg/L (ref)	0	0	0	0	0	-
1.0–1.5 mg/L	−0.421	0.248	0.656	0.403	1.068	0.090
≥1.5 mg/L	−0.769	0.305	0.463	0.255	0.842	<0.05
Seminal Pb						
0 μg/L (ref)	0	0	0	0	0	-
0–10 μg/L	0.511	0.224	1.666	1.074	2.584	<0.05
≥10 μg/L	0.790	0.340	2.204	1.132	4.291	<0.05

^1^ CI = confidence interval. ^2^ Ref = reference. ^3^ “0” represented the reference category for each explanatory variable.

**Table 8:** Prediction of the occurrence probability of boar abnormal sperm morphology less than 10% or greater than 20% under different breeds and element concentrations.

Breed	Subgroups	P (Abnormal Rate ≤ 10%)	P (Abnormal Rate > 20%)
Duroc	Serum Cu ≤ 2.0 mg/L	19.37%	19.50%
	Serum Cu ≥ 2.5 mg/L	10.71%	32.67%
	Serum Fe ≤ 1.0 mg/L	19.37%	19.50%
	Serum Fe ≥ 1.5 mg/L	34.14%	10.09%
	Seminal Pb = 0 μg/L	19.37%	19.50%
	Seminal Pb = 0–10 μg/L	12.60%	28.76%
	Seminal Pb ≥ 10 μg/L	9.83%	34.80%
	Mean value	17.91%	23.55%
Yorkshire	Serum Cu ≤ 2.0 mg/L	42.31%	7.35%
	Serum Cu ≥ 2.5 mg/L	26.80%	13.72%
	Serum Fe ≤ 1.0 mg/L	42.31%	7.35%
	Serum Fe ≥ 1.5 mg/L	61.28%	3.55%
	Seminal Pb = 0 μg/L	42.31%	7.35%
	Seminal Pb = 0–10 μg/L	30.56%	11.68%
	Seminal Pb ≥ 10 μg/L	24.97%	14.88%
	Mean value	38.65%	9.41%
Landrace	Serum Cu ≤ 2.0 mg/L	50.42%	5.41%
	Serum Cu ≥ 2.5 mg/L	33.67%	10.28%
	Serum Fe ≤ 1.0 mg/L	50.42%	5.41%
	Serum Fe ≥ 1.5 mg/L	68.70%	2.58%
	Seminal Pb = 0 μg/L	50.42%	5.41%
	Seminal Pb = 0–10 μg/L	37.90%	8.71%
	Seminal Pb ≥ 10 μg/L	31.58%	11.19%
	Mean value	46.16%	7.00%
